# The effects of stigma and social support on the health-related quality of life of people with drug resistance tuberculosis in Lagos, Nigeria

**DOI:** 10.1007/s11136-025-03902-5

**Published:** 2025-02-18

**Authors:** Olusola Adedeji Adejumo, Champaklal Jinabhai, Olusoji Daniel, Firoza Haffejee

**Affiliations:** 1 Department of Basic Medical Sciences, Durban University of Technology, Durban, South Africa; 2 Mainland Hospital Yaba, Lagos, Nigeria; 3 Faculty of Health Sciences, Durban University of Technology, Durban, South Africa; 4 Department of Community Medicine and Primary Care, Olabisi Onabanjo University, Sagamu, Nigeria

**Keywords:** Health-related quality of life, TB-stigma, Social support, Nigeria

## Abstract

**Purpose:**

This study assessed the effects of TB stigma and social support on the health-related quality of life (HRQoL) of people living with drug-resistant tuberculosis (DR-TB) in Lagos, Nigeria.

**Methods:**

A cross-sectional study was conducted in five DR-TB treatment centres in Lagos, Nigeria, between September and December 2023. A total of 203 adults on DR-TB treatment were recruited to complete a questionnaire including the Redwood DR-TB stigma scale, the Functional Assessment of Chronic Illness Therapy-TB (FACCIT) scale, and the Multidimensional Scale of Perceived Social Support (MSPSS). Student ‘t’ test/one-way ANOVA, Pearson’s correlation, and hierarchical linear regression analysis were conducted to explore the factors associated with HRQoL and the relationships between stigma, social support, and HRQoL.

**Results:**

The mean overall HRQoL was 41.1 ± 12.9 among people with DR-TB. The HRQoL score of the physical domain was the lowest (25.8 ± 13.8). Participants who were young, male, single, with higher education, and HIV-negative had higher HRQoL than their counterparts (p < 0.05). Stigma was negatively associated with HRQoL, while social support was positively related, collectively explaining 57.6% of the variance. In the final model, social support contributed more (B = 0.576) to predicting HRQoL than did stigma (B = − 0.414).

**Conclusion:**

The overall HRQoL of people with DR-TB in Lagos, Nigeria, was poor. Strategies that improve social support systems and reduce stigma are needed to improve this. Further studies are also required to assess the changes in HRQoL over time and evaluate the impact of specific stigma-reduction interventions.

## Plain English summary

There is a renewed interest in measuring the health-related quality of life of people with long-standing diseases because it measures the effect of diseases on regular activities and functions. This concept has been studied among people with tuberculosis. Several studies from Nigeria have assessed the factors associated with the health-related quality of life among people with tuberculosis, but little is known about the effect of stigma and social support on the health-related quality of life of people with tuberculosis in Lagos, Nigeria. We conducted a study among people receiving treatment for resistant tuberculosis in Lagos, Nigeria, and assessed the effect of stigma and social support on their health-related quality of life. Our study suggested that people with resistant tuberculosis's overall health-related quality of life was generally poor. However, the younger participants, males, those with higher education, and who were HIV-negative, had higher health-related quality of life than their counterparts. The greater the stigma experienced by our participants, the lower the health-related quality of life recorded. Conversely, the more social support received, the higher the health-related quality of life. In conclusion, our study suggested that strategies to reduce stigma and strengthen social support are needed to improve the health-related quality of life of people with resistant tuberculosis in Lagos, Nigeria.

## Introduction

Health-related quality of life (HRQoL) assesses the impact of disease and its treatment on the person’s daily perception of physical, mental and social well-being [1]. It is an essential concept because people with chronic diseases often prioritise their cognitive and social well-being the same way they do their physical health [2]. Patients are not concerned about bacteriological cures alone; their well-being, functional capacity, and illness experiences are equally important [3]. It is an important patient-reported outcome measure as clinicians could underestimate the impacts of disease and treatment on HRQoL [4].

HRQoL applies a broader concept of health status measurement beyond the usual mortality and morbidity indicators. It has become an area of interest to stakeholders because it measures the effect of diseases and outcomes on regular activities and functions [5]. Tuberculosis (TB) is one of the diseases that can adversely undermine the HRQoL [6] and can be influenced by several patient, disease and treatment-related factors in people with TB (PWTB) because of the multi-drug therapy, side effects of medications, social impacts, social support, social stigma and possible complications [7, 8]. Generally, HRQoL is lower in PWTB compared with healthy populations [9, 10] but higher among people with drug-sensitive TB compared with their counterparts with drug-resistant TB (DR-TB) due to a complex interaction between physical illness, psychological consequences of the disease and the financial burden [11].

Stigma is a global public health challenge and a significant factor affecting TB control globally [12]. Crucially, it is a barrier to achieving the World Health Organization (WHO) goal of ending TB by 2050 [12]. Deep-rooted myths, wrong perceptions and beliefs about TB and those affected are factors responsible for stigma at the family and community level [13, 14]. Goffman described stigma as an “attribute that is deeply discrediting that demeans people from a whole and usual person to a tainted, discounted one” [15]. Stigma weakens TB response and damages the lives and health of those who experience it [16]. The effects of TB stigma are far-reaching. Unfortunately, necessary attention has not been given to international TB prevention and control [17].

Social support is the perceived or actual care received from family, friends, and significant others [18]. It buffers adverse life events and improves TB treatment outcomes [19]. When adequate, social support improves self-confidence and life fulfilment, reducing psychological distress [20]. There is an intricate relationship between TB stigma, social support, and HRQoL. Studies have shown that people with TB who experienced a lower level of stigma had good HRQoL; however, in contrast, social support has the opposite effect [21, 22]. There is, however, a lack of studies that accessed which factor between stigma and social support made a significant contribution to predicting HRQoL in people with TB.

Nigeria is classified as a high-burden TB, TB/HIV and drug-resistant TB (DR-TB) country by the WHO, with an estimated incidence rate of 219 per 100,000 population [19]. In 2022, about 43% of the 408,500 DR-TB cases were enrolled globally. Nigeria and nine other countries (eight in Asia and one in Europe) accounted for 70% of the treatment initiation gap [23]. Lagos is a cosmopolitan state with an estimated population of about 21 million, accounts for 8.4% of the national TB burden and contributes 9.4% of the country’s total TB notification [24]. As the country's commercial nerve centre, Lagos State has unique socio-cultural dynamics because of ethnic diversity. The health system challenges and the numerous slums (over 100) in the state reflect its mega-city status [25]. It was the first state to decentralise DR-TB treatment to the community because of its high population density and poor physical access to DR-TB treatment centres.

TB is a stigmatised disease and a formidable challenge to global TB control [12]. A high prevalence of TB stigma has been reported in Nigeria and other high-burdened TB countries [26–28]. HRQoL has been studied among people with drug-sensitive and drug-resistant TB in Nigeria. These studies focused on the effects of TB treatment on HRQoL and the socio-demographic and clinical factors associated with HRQoL [29–32]. There is a need to assess the impact of TB stigma and social support on the HRQoL of PWTB in Nigeria because of the burden of TB and the high prevalence of TB stigma in the country. Unfortunately, there is little evidence about the effect of stigma and social support on HRQoL among DR-TB patients receiving treatment. This present study assessed the effect of TB stigma and social support on the HRQoL and which factor contributed more to predicting HRQoL among people living with DR-TB in Lagos, Nigeria. We envisage that our findings will contribute to the call for integrated patient-centred care among people with DR-TB, which is one of the pillars of the End TB Strategy.

## Methods

*Study design and sampling*: A descriptive cross-sectional study was conducted between September and December 2023 to assess the HRQoL of people with drug-resistant TB in Lagos, Nigeria, and determine the effect of TB stigma and social support. Of the six DR-TB centres in Lagos, five centres with high caseloads of people with DR-TB enrolled for care were purposely selected for the study to ensure geographic representation and diversity in patient demographics.

Convenient sampling was used. Eligible participants willing to participate in the study were consecutively recruited. Enrollment in the study was done during the outpatient clinic. All study participants signed a written informed consent form before the research assistants administered the questionnaire.

Using the sample size formula for a finite population, the TB stigma prevalence rate of 18%, [20] a non-response rate of 15% and the average population of 625 people with DR-TB on treatment in Lagos (from the records of the Lagos State TB and Leprosy control program), a sample size of 200 was obtained. Two hundred and three participants were recruited for the study.

*Eligibility criteria*: Participants were recruited into the study if they were adults (18 years and above), diagnosed with Xpert MTB/RIF assay and had been on DR-TB treatment for at least eight weeks.

*Exclusion criteria*: People with DR-TB who were very ill, who didn’t understand English, and those who were unable to make an informed decision on participation in the study due to physical, cognitive or psychological factors were excluded from the study.

### Measurement of HRQoL

The HRQoL was measured in this study using the Functional Assessment of Chronic Illness Therapy-TB (FACIT) scale [33]. The FACIT scale has been used to measure HRQoL in the general population and chronic diseases such as HIV/AIDs, multiple sclerosis, Parkinson's disease, and rheumatoid arthritis. It is widely used and translated into over 45 languages [34] The FACIT Measurement System provides an array of generic and targeted measures. It has five subscales: physical, social, emotional, functional and spiritual. The health concepts described by the scale range in score from 0 to 188 (for the 47-item FACIT-TB), with higher scores indicating higher levels of function and better health. Scores obtained for each domain were transformed to 100 since each domain does not contain an equal number of questions. The participants’ responses are presented as a profile of scores calculated for each sub-scale. The overall score was calculated by summing up all the sub-scale scores [33]. The Cronbach’s alpha coefficient for the FACIT scale in this study was 0.825.

### Measurement of stigma

The Redwood DR-TB stigma scale was used to measure experienced stigma termed TB stigma in this study. It is a 14-item scale scored on a 3-point Likert scale ranging from 0 (strongly disagree) to 3 (strongly agree). The score ranged from 0 to 42, and a higher score indicates a higher level of stigma. TB stigma is measured across four factors: guilt, social exclusion, physical isolation, and blame [35]. The sum of all the subscale scores refers to experiencing TB stigma in this study. The Redwood DR-TB stigma scale was specifically designed to assess the stigma experienced by people living with DR-TB and also aid in the evaluation of stigma reduction scales, unlike other scales [35]. The Redwood DR-TB stigma scale has a Cronbach’s alpha coefficient of 0.725 in this study.

### Measurement of social support

We measured social support using a 12-item Multidimensional Scale of Perceived Social Support (MSPSS). The scale is scored on a 7-point Likert Scale ranging from 1 (very strongly disagree) to 7 (very strongly agree) [36]. The MSPSS addresses the subjective assessment of the adequacy of social support from three perspectives: family, friends, and significant others. It is psychometrically sound and has good reliability and adequate construct validity. It is self-explanatory, simple to use and time-conserving, making it an ideal tool for participants with limited time [36]. The scores ranged from 1 to 84, and higher scores indicated a high perceived level of social support. In this study, Cronbach’s alpha coefficient for MSPSS was 0.780.

### Measures

*Outcome measure*: In this study, the outcome measure is HRQoL, measured as a continuous variable.

*Independent measures*: Age, gender, marital status, educational status, working status, HIV status, being a breadwinner, barrier to treatment, cigarette smoking and alcohol intake, TB stigma and social support.

*Data collection*: Data collection was done using a questionnaire divided into two sections. The first section collected sociodemographic details of participants, HIV status, intake of cigarettes and alcohol and barrier to treatment. The three (stigma, social support and HRQoL) tools described above were combined in the second part of the questionnaire. Five trained research assistants administered the questionnaire. The tools were piloted in the treatment facility not recruited for the study. Modifications were made to the tools to suit the local context and improve participants' acceptability and understanding after the pilot study. The validity and internal consistency of the scales were assessed using the Cronbach alpha.

### Ethical considerations

The Institutional Research Ethic Committee of Durban University of Technology, South Africa (IREC 066/230, 20th September 2023) and the Health Research and Ethics Committee of the Lagos State University Teaching Hospital (LREC/06/10/2179, 20th June 2023) gave ethical approval for this study. The Lagos State Ministry of Health and the medical directors of the health facilities where participants were recruited permitted the participants’ interview. Participants who were stigmatised and had poor quality of life were referred for counselling by a psychologist, which was at no cost to the participants.

### Statistical analyses

The HRQoL scores of the different subscales were standardised before being summed up. In this study, a test for normality was conducted for the outcome variable (HRQoL). Continuous variables such as HRQoL and age were represented as mean and standard deviation. Categorical variables were expressed as percentages. The Student's t-test and one-way ANOVA were used to compare the HRQoL subscale scores with different demographic and clinical parameters. The effect size for the Student's t-test and one-way ANOVA was measured using Hedges’g and eta squared (ƞ_p_^2^). For eta squared 0.010–0.050 graded as small, 0.060–0.138, medium and > 0.138, large while Hedges’g classified effect sizes as small (0.2), medium (0.5) and large (≥ 8) [37, 38]. Pearson’s correlation analysis assessed the correlation between HRQoL, TB stigma and social support. Hierarchical multiple regression was applied to determine the effects of influencing factors on HRQoL. Preliminary analyses showed no violation of the assumptions of normality, linearity, multicollinearity (variance inflation factor was < 10 and > 0.1) and homoscedasticity. In step 1, age, gender, marital status, educational status and HIV status (which were associated with HRQoL in univariate analysis) were entered in step 1. Stigma was added in step 2, while social support was added in step 3. The IBM Statistics Version 26 (IBM Corporation, Armonk, State of New York) for statistical analysis was used for the statistical analyses. A two-tailed p-value of < 0.05 was considered statistically significant.

## Results

### Socio-demographic associations

Two hundred and three participants were recruited for the study with a mean age of 39.2 ± 13.8 years. There was no difference in the mean age of the males (40.1 ± 13.6) and females (37.8 ± 14.1) p = 0.256. A higher proportion of the males were breadwinners (63.3% vs 24.0%, p < 0.001), smoked cigarettes (35.9% vs 5.3%, p < 0.001), and took alcohol (54.7% vs 20.0%, p < 0.001) compared to the females. More females than males were HIV positive (19 ± 26.4 vs 16 ± 13.0, p < 0.019; Table 1).

**Table 1:** Socio-demographic and clinical details of participants

Variable	Total	Male	Female	p
	n = 203 (%)	n = 128 (%)	n = 75 (%)	
*Age group (years)*				
< 30	54 (26.6)	30 (23.4)	24 (32.0)	0.256
30–59	128 (63.1)	83 (64.8)	45 (60.0)	
≥ 60	21 (10.3)	15 (11.7)	6 (8.0)	
Age—mean (SD)	39.2 (13.8)	40.1 (13.6)	37.8 (14.1)	
*Marital status*				
Single	74 (36.5)	44 (34.4)	30 (40.0)	**0.033**
Married	111 (54.7)	77 (60.2)	34 (45.3)	
Widowed/separated	18 (8.9)	7 (5.5)	11 (14.7)	
*Educational status*				
No education/primary	43 (21.2)	28 (21.9)	15 (20.0)	0.910
Secondary	101 (49.8)	64 (50.0)	37 (49.3)	
Tertiary	59 (29.1)	36 (28.1)	23 (30.7)	
*Working status*				
Working	105 (51.7)	71 (55.5)	34 (45.3)	0.163
Not working	98 (48.3)	57 (44.5)	41 (54.7)	
*Bead winner*				
Yes	99 (48.8)	81 (63.3)	18 (24.0)	**< 0.001**
No	104 (51.2)	47 (36.7)	57 (76.0)	
*HIV status*				
Positive	35 (17.2)	16 (13.0)	19 (26.4)	**0.019**
Negative	160 (78.8)	107 (87.0)	53 (73.6)	
Unknown^#^	8 (3.9)	5 (3.9)	3 (4.0)	
*Barriers to TB treatment*				
Yes	36 (17.7)	22 (17.2)	14 (18.7)	0.790
No	167 (82.3)	106 (82.8)	61 (81.3)	
*Smoked cigarette*				
Yes	50 (24.6)	46 (35.9)	4 (5.3)	**< 0.001**
No	153 (75.4)	82 (64.1)	71 (94.7)	
*Alcohol intake*				
Yes	85 (41.9)	70 (54.7)	15 (20.0)	**< 0.001**
No	118 (58.1)	58 (45.3)	60 (80.0)	

Bold values indicate p-value < 0.05 are considered significant

^#^Not part of the analysis

### HRQoL subscales and associations

The average overall HRQoL score was 41.1 ± 12.9. The average physical, social, emotional, functional and spiritual subscale scores were 25.8 ± 13.8, 61.2 ± 13.1, 36.7 ± 12.1, 50.8 ± 14.9 and 74.3 ± 15.9 respectively (Table 2).

**Table 2:** Participants Health-related Quality of life domain scores

Variable	n	Mean (SD)	95%CI
Overall HRQoL score	203	41.1 (12.9)	39.3–42.8
Physical	203	25.8 (13.8)	23.9–27.7
Social	203	61.2 (13.1)	58.4–64.0
Emotional	203	36.7 (12.1)	34.3–39.1
Functional	203	50.8 (14.9)	47.8–53.8
Spiritual	203	74.3 (15.9)	71.3–77.3

The factors associated with the overall HRQoL and its subscales are shown in Table 3. The overall HRQoL scores were significantly higher among male participants, those below 30 years, who were single and with tertiary education than their counterparts (p < 0.05). Also, the overall HRQoL is associated with educational status, employment and HIV status (p < 0.05). Participants under 30 had a significantly higher HRQoL score in the physical, emotional and function subscales. Except for the social subscale, where the males had a higher HRQoL score than the females, there was no gender difference in the HRQoL scores in other subscales. Participants with tertiary education had higher HRQoL scores in the physical, social, emotional and functional subscales. Being HIV positive was associated with lower HRQoL scores in the physical and social subscale. Also, the physical, social, emotional and functional HRQoL scores were lower among widows/widowers and separated than the singles and married. (Table 3).

**Table 3:** Health-related quality of life scores

Variable	Physical	Social	Emotional	Functional	Spiritual	Total HRQOL
	Mean (SD), effect size	Mean (SD), effect size	Mean (SD), effect size	Mean (SD), effect size	Mean (SD), effect size	Mean (SD), effect size
*Age group (years)*						
< 30	29.4 (15.9)*, 0.034	63.2 (20.7), 0.006	43.3 (18.2)*, 0.055	57.7 (22.5)*, 0.040	78.1 (19.7), 0.016	45.6 (14.5)*, 0.054
30–59	25.1 (12.5)	61.0 (20.2)	34.7 (16.4)	49.4 (21.6)	73.8 (22.7)	40.1 (12.4)
≥ 60	20.6 (6.5)	57.7 (18.5)	32.5 (14.1)	42.7 (18.5)	68.3 (21.8)	35.9 (7.4)
*Gender*						
Male	26.9 (14.5), 0.221	64.2 (20.2)*, 0.410	38.1 (18.0), 0.212	52.1 (22.6), 0.154	76.3 (21.9), 0.242	42.7 (13.7)*, 0.327
Female	23.9 (12.4)	56.1 (19.1)	34.4 (15.3)	48.7 (20.7)	71.0 (21.8)	38.5 (11.1)
*Marital status*						
Single	28.5 (14.4)*, 0.036	62.4 (20.8)*, 0.047	41.6 (19.6)*, 0.064	55.0 (24.2)*, 0.037	78.8 (18.4), 0.029	44.4 (14.1)**, 0.072
Married	25.0 (13.9)	62.7 (19.6)	35.1 (15.0)	49.8 (20.3)	72.5 (23.1)	40.4 (12.0)
Widowed/ separated	19.3 (7.2)	47.2 (15.7)	27.0 (12.1)	39.7 (17.2)	67.1 (25.3)	31.9 (7.3)
*Educational status*						
No educ/primary	22.4 (10.9)**, 0.081	54.7 (22.6)*, 0.042	29.4 (14.6)**, 0.090	44.1 (20.5)*, 0.039	69.8 (24.7), 0.015	35.7 (12.1)**, 0.097
Secondary	23.7 (11.9)	61.0 (19.2)	35.7 (15.9)	50.4 (21.0)	76.6 (20.9)	40.1 (10.4)
Tertiary	31.9 (16.9)	66.3 (18.5)	43.8 (18.4)	56.4 (23.4)	73.9 (21.4)	46.8 (15.2)
*Working condition*						
Presently working	28.3 (15.7)* 0.382	63.0 (20.6), 0.185	36.5 (17.8), 0.025	56.1 (22.9)**, 0.516	74.2 (23.3), 0.013	43.1 (14.5)*, 0.323
Not working	23.1 (10.9)	59.3 (19.5)	37.0 (16.4)	45.1 (19.4)	74.5 (20.4)	39.0 (10.5)
*Bread winner*						
Yes	23.9 (12.1), 0.262	59.2 (20.2), 0.197	34.1 (16.3)*, 0.299	49.5 (20.8), 0.113	72.6 (24.2), 0.151	39.1 (12.0)*, 0.298
No	27.6 (15.2)	63.1 (19.9)	39.2 (17.6)	52.0 (22.9)	76.0 (19.6)	42.9 (13.5)
*HIV status*						
Positive	19.6 (8.8)**, 0.576	54.5 (19.8)*, 0.441	34.4 (14.5), 0.163	45.2 (21.8), 0.303	70.5 (24.4), 0.206	36.0 (10.8)*, 0.493
Negative	27.4 (14.4)	63.3 (20.0)	37.1 (17.6)	51.7 (21.4)	75.1 (21.5)	42.3 (13.2)
Unknown^#^	20.0 (12.2)	49.1 (15.8)	39.2 (18.5)	56.7 (21.4)	77.1 (18.8)	38.7 (10.1)
*Barrier to TB*						
Yes	23.4 (12.5), 0.213	57.8 (19.7), 0.204	33.5 (13.9), 0.228	47.3 (21.2), 0.193	65.7 (24.0)*, 0.483	37.7 (11.3), 0.316
No	26.3 (14.1)	62.0 (20.2)	37.4 (17.7)	51.6 (22.0)	76.2 (21.1)	41.8 (13.1)
*Ever smoked*						
Yes	26.9 (15.0), 0.106	62.3 (21.4), 0.070	37.8 (18.0), 0.083	48.4 (22.6), 0.143	75.2 (24.4), 0.049	41.6 (13.5), 0.054
No	25.4 (13.5)	60.9 (19.8)	36.4 (16.8)	51.6 (21.7)	74.1 (21.2)	40.9 (12.7)
*Alcohol intake*						
Yes	26.5 (13.7), 0.087	63.2 (21.0), 0.168	38.2 (17.1), 0.149	51.6 (21.3), 0.058	75.2 (22.6), 0.066	42.2 (13.0), 0.148
No	25.3 (14.0)	59.8 (19.4)	35.7 (17.1)	50.3 (22.4)	73.7 (21.6)	40.3 (12.8)
*Length of treatment*						
1–2 months	25.8 (13.4), 0.000	63.6 (20.1), 0.008	36.8 (17.1), 0.000	46.2 (20.7), 0.025	73.3 (22.4), 0.008	40.7 (12.7), 0.001
3–4 months	25.7 (14.4)	61.3 (18.7)	36.4 (18.3)	51.1 (22.8)	74.1 (22.0)	41.1 (13.7)
≥ 5 months	25.9 (14.0)	59.3 (21.1)	36.9 (16.4)	54.3 (21.8)	75.3 (21.8)	41.5 (12.7)

NB: *p < 0.05, **p < 0.001, ^#^Not part of the analysis, *SD* standard deviation

### Relationship between HRQoL, social support and stigma

**Fig. 1:**
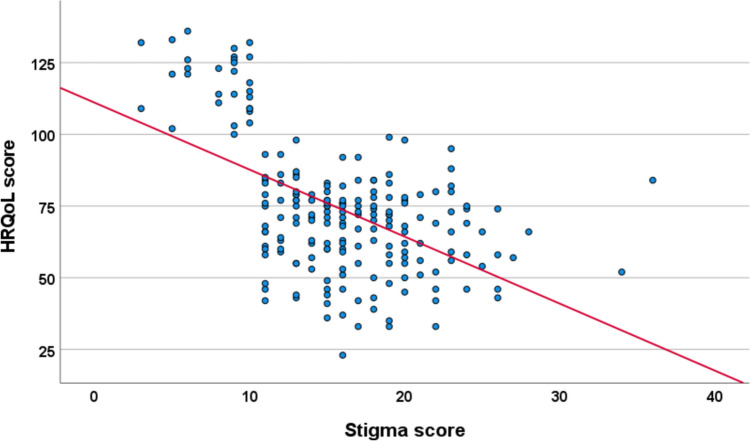
The relationship between HRQoL and stigma

**Fig. 2:**
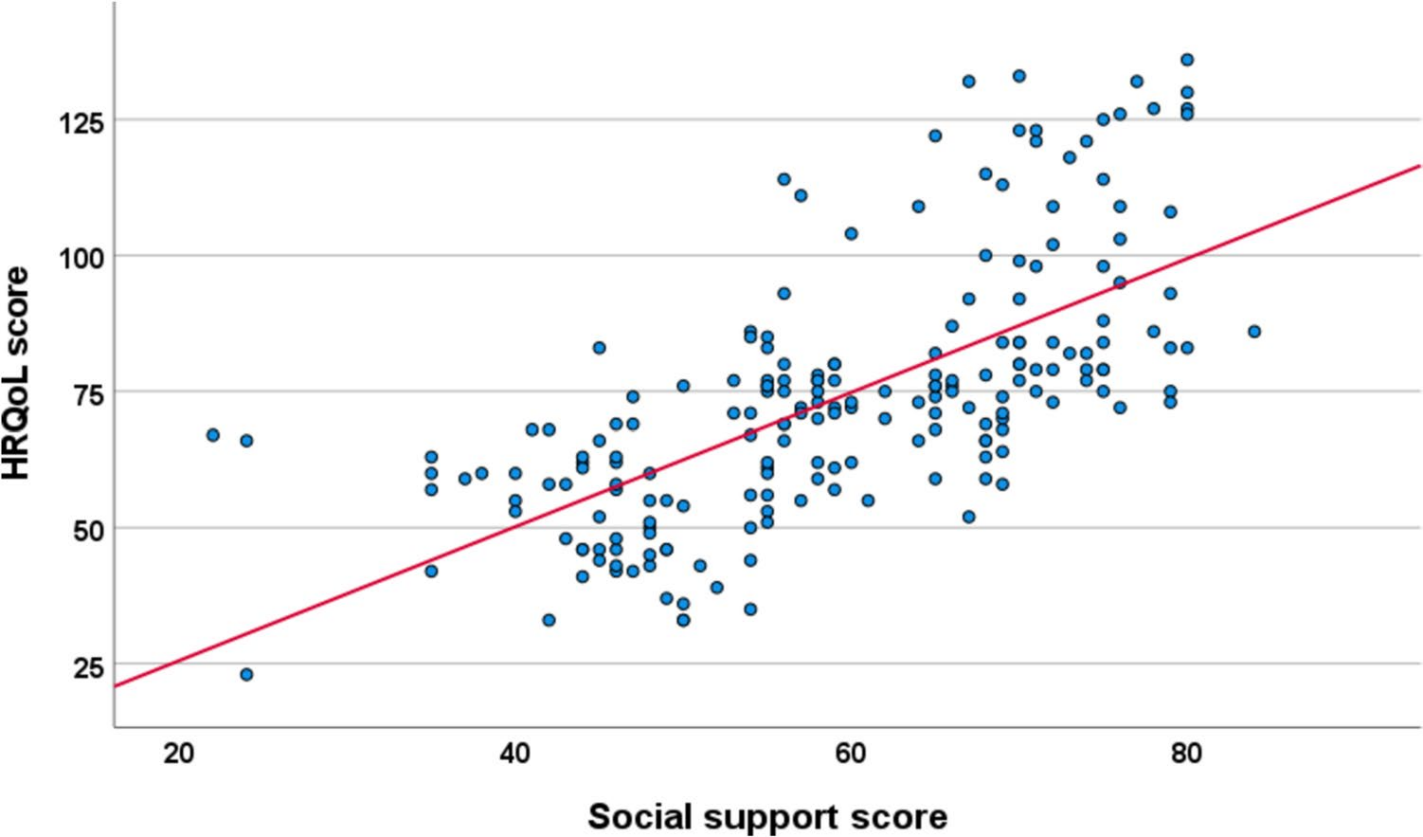
The relationship between HRQoL and social support

The correlation between the overall HRQoL scores and TB stigma and social support is shown in Table 4. The overall HRQoL was negatively correlated with the TB stigma (r = − 0.536, p < 0.001) and positively correlated with social support (r = 0.416, p < 0.001). TB stigma was negatively correlated with social support (r = − 0.700, p < 0.001). Figure 1 shows an increase in HRQoL as stigma decreases, implying a negative correlation. Also, HRQoL increases as social support increases (positive correlation), as shown in Fig. 2.

**Table 4:** Scores and correlation of total HRQOL with stigma and social support

Variable	Mean (SD)	95%CI	Total HRQoL score	Stigma score	Social support score
Total HRQOL score	41.1 (12.9)	39.3–42.9	1	− 0.536**	0.416**
Stigma Score	15.9 (5.3)	15.1–16.6	− 0.536**	1	− 0.700**
Social support score	59.3 (12.6)	57.6–61.1	0.416**	− 0.700**	1

NB: Pearson’s correlation analysis was used to analyze the correlation among HRQoL, TB stigma, social support

*HRQoL* Health related quality of life, *SD* Standard deviation

**p < 0.01

### Hierarchical regression analysis

Table 5 contains the hierarchical regression analysis of the overall HRQoL. The independent variables associated with the overall HRQoL in univariate analysis (p < 0.05) were entered into the hierarchical multiple regression. In step 1, the combination of age, gender, marital status and HIV status accounted for 3.3% of the variance in HRQoL (F = 1.274, R^2^ = 0.033, p = 0.277). In step 2, after controlling for the demographic variables, TB stigma was negatively associated with HRQoL (B = − 0.520, p < 0.001) but accounted for 26.4% variance in HRQoL (F = 13.215, adjusted R = 0.297, ΔR^2^ = 0.264, p < 0.001). In step 3, social support was added to the model and was positively associated with HRQoL (B = 0.578, p < 0.001). Social support accounted for 31.2% of the variance in HRQoL (F = 41.463, R^2^ = 0.608, ΔR^2^ = 0.312, p < 0.001). All the variables in the model explained 60.8% of the variance in HRQoL. Social support made a higher significant contribution (B = 0.578, p < 0.001) in predicting HRQoL than stigma (B = − 0.415, p < 0.001). In the final model, social support contributed more (B = 0.576) to predicting HRQoL than stigma (B = − 0.414).

**Table 5:** Hierarchical multiple regression analysis results of HRQoL

Variable	Step 1		Step 2		Step 3	
	B	p-value	B	p-value	B	p-value
Age	− 0.052	0.519	− 0.036	0.594	− 0.018	0.718
Gender	− 0.035	0.633	− 0.028	0.652	− 0.051	0.278
Marital status	− 0.160	0.047	− 0.121	0.080	− 0.027	0.601
Educational status	0.086	0.234	0.102	0.099	0.036	0.437
HIV status	− 0.087	0.239	− 0.011	0.174	− 0.014	0.767
Stigma			− 0.520	**< 0.001**	− 0.415	**< 0.001**
Social support					0.578	**< 0.001**
F	1.274	0.277	13.215	**< 0.001**	41.463	**< 0.001**
R^2^	0.033		0.297		0.608	
Adjusted R^2^	0.007		0.274		0.593	
ΔR^2^	0.033		0.264		0.312	

Bold values indicate p-value < 0.05 are considered significant

## Discussion

This study assessed the effects of TB stigma and social support on HRQoL among people with DR-TB on treatment in Lagos, Nigeria. In this study, the overall HRQoL score was poor. The physical and emotional subscales had the lowest HRQoL score, while the spiritual subscale had the highest score. Age, gender, marital status, educational status and HIV status were factors associated with the overall HRQoL. The duration of treatment was not associated with HRQoL. Stigma was negatively associated with HRQoL, while social support was positively associated with HRQoL. Social support contributed more to predicting HRQoL than stigma.

The overall low HRQoL score of participants in this study was similar to cross-sectional studies from China, Eritrea and India, where the HRQoL was significantly reduced among people with DR-TB [11, 39, 40]. Contrary to our findings, a study from Nigeria reported high HRQoL among people with DR-TB [31]. However, the above report did not state the duration of treatment of participants. Furthermore, different tools used to measure HRQoL and sampling variation may be responsible for this finding. People with DR-TB have difficulties related to their family life, social stigmatisation and financial hardship due to longer treatment duration, toxic medications, frequent adverse effects and associated poorer outcomes, consequently impairing their HRQoL [41]. The poor socio-economic conditions of people with DR-TB, the severity of the disease, the financial burden of the disease, and inadequate access to DR-TB healthcare services in Nigeria may also be responsible for our findings [11, 42].

Our study showed that the HRQoL score was highest in the spiritual subscale. A systemic review showed a positive association between spirituality/religiousness and the quality of life of adults [43]. Nigerians are very religious, and over 99% identify with one religion. The cultural settings of the ethnic groups in the country also shape people’s belief systems [44]. Most individuals turn to their faith when faced with health challenges, which sometimes could be detrimental to their health. Understanding how religiousness influences HRQoL could help develop patient-centred care in culturally diverse settings like Nigeria.

The effects of DR-TB treatment on HRQoL are variable. According to a study from Yemen, DR-TB treatment influenced the increase in HRQoL scores of people with DR-TB in the late phases of treatment [10]. Contrary to this, our study shows that the HRQoL did not increase with the duration of treatment. A follow-up study from Pakistan showed a minimal clinically insignificant improvement in the HRQoL score after one year of DR-TB treatment [1]. The reason for this is unknown; the variations in the different study populations' cultural, socio-economic and social support systems may be contributory factors. The physical, emotional, and functional domain HRQoL scores in this study were poor, irrespective of the duration of treatment, similar to the findings from India [40].

In the assessment of the HRQoL scores among participants, we found that age had a significant effect on the overall HRQoL. The overall HRQoL was higher among the younger than the older age group. This conforms with previous studies from China, India and Nigeria [39, 45, 46]. The effects of advancing age, economic difficulties, poor mobility, and comorbidities, common among older people, could be responsible for this finding [47]. The younger participants were more likely to have better financial resources to access care, have good social relationships, have a better understanding of the disease process and have better self-esteem than the older participants [46]. Males had a higher score in the overall HRQoL than females in our study, consistent with findings from India [48, 49]. The patriarchal nature of Nigerians causes gender inequalities, which made the females the weaker sex, marginalised, discriminated against, and economically dependent on men and the cultural perceptions that relegate women to the background, without any opinion of their own, all of which potentially cause poor expression of views and feelings, may be responsible for the poorer HRQoL among women [46, 50]. Similar to our findings, studies have opined that a higher HRQoL was found among people with DR-TB who had a higher education compared to their counterparts [39, 51]. Education enables people to be well-informed, have better social connections, and understand the disease process, invariably improving their HRQoL [16]. Comorbidity has a powerful influence on HRQoL [39]. HIV-positive participants had poorer HRQoL than HIV-negative participants in this study, consistent with findings from other studies [52, 53]. TB and HIV co-infection are associated with unique therapeutic challenges and often pose a burden on the health system. Both diseases combine to impose physical and mental distress, leading to poor disease outcomes and poorer HRQoL [53].

One of the most critical factors affecting HRQoL among people with DR-TB is stigma. In our study, stigma alone explained more than a third of the variance in HRQoL. In agreement with other studies from China, India, Indonesia and Singapore [21, 22, 54, 55], our analysis shows that stigma was negatively associated with HRQoL. A systematic review from South Africa evaluating HRQoL among PWTB suggested that psycho-social burden such as stigma and social isolation impacts HRQoL more than clinical symptoms [56]. TB is a stigmatised disease because of fear of infection, its association with HIV disease and traditional myths [17]. TB stigma predisposes to depression, low self-efficacy, anxiety and ultimately poor HRQoL [57]. The prevention of TB stigma is crucial to global TB control because of its adverse effects on health-seeking behaviour and disease outcomes [58]. TB stigma can occur in the family, community, health facility and organisation. It may result in rejection, social exclusion, loss of employment, divorce and rejection of marriage proposals. This discrimination may result in mental distress and poor HRQoL [59]. Contrary to general belief, stigmatisation of people with TB is not limited to developing countries; it has also been reported in other low-burden TB countries [60].

There is a lack of studies evaluating interventions to reduce TB stigma despite its global importance. The public, healthcare workers managing TB patients, and other healthcare workers are possible sources of TB stigma. Interventions are optimised when simultaneously targeted towards more than one key population [61]. Nigeria can leverage TB stigma reduction interventions such as public awareness campaigns, stakeholder consultations, counselling, and peer support groups in high-burden settings [62]. Researching which intervention works best in a culturally diverse setting like Lagos could be the starting point.

For decades, social support has been recognised as an essential element in the control of TB [63]. In this study, social support was positively associated with the HRQoL of people with DR-TB, consistent with reports from India and Zimbabwe [40, 64]. Patients with high social support are more likely to initiate diagnosis and treatment early [65], comply with treatment regimens [56], and experience less stigma [56, 57], ultimately leading to better HRQoL [66]. Social support includes support from family, friends, the community, and the health institution. When these are inconsistent, it is suggestive of societal stigma [64]. Institutional support from implementing partners and government agencies [31] and support in the workplace from healthcare providers and families positively influence the HRQoL of people with DR-TB [40]. Social support and TB stigma had opposite effects on HRQoL. However, social support impacted the HRQoL more than stigma in this study, similar to the findings from Pakistan [67]. Many studies have documented the effects of stigma and social support on HRQoL among people with TB; there is a need for further studies to ascertain which of the factors have a higher impact on HRQoL.

This study has some limitations. First, a causal relationship between TB stigma, social support, and HRQoL cannot be drawn from our research, as it is a cross-sectional study. Second, there is a possibility of having a response and ascertainment bias in the study because the measurements were self-reported.

## Conclusion

This current study has established that the HRQoL of people with DR-TB in Lagos, Nigeria, is poor. Improvement in social support and reduction in stigma experienced by people with DR-TB will improve their HRQoL. The social support structure in the DR-TB programme needs to be evaluated and strengthened. There is also a need to encourage social support at the family and community levels. In contrast, policies that will reduce discrimination and stigma against people with DR-TB in the community, among healthcare providers and public health agencies, are urgently needed. Further studies are required to establish these associations, assess the changes in HRQoL over time and evaluate the impact of specific stigma-reduction interventions. Addressing the issue of stigma may facilitate the achievement of the prohibition of stigma and discrimination against people with TB, which is one of the thrusts of the End TB strategies.

## Data Availability

The data underlying this article cannot be shared publicly due to the privacy of individuals who participated in the study. The data will be shared on reasonable request to the corresponding author.
